# Digital Pain Mapping and Tracking in Patients With Chronic Pain: Longitudinal Study

**DOI:** 10.2196/21475

**Published:** 2020-10-26

**Authors:** Maria Galve Villa, Thorvaldur S Palsson, Albert Cid Royo, Carsten R Bjarkam, Shellie A Boudreau

**Affiliations:** 1 Center for Neuroplasticity and Pain, Center for Sensory Motor Interaction, Department of Health Science and Technology Faculty of Medicine Aalborg University Aalborg Denmark; 2 Center for Sensory Motor Interaction, Department of Health Science and Technology Faculty of Medicine Aalborg University Aalborg Denmark; 3 Department of Neurosurgery Institute of Clinical Medicine Aalborg University Hospital Aalborg Denmark

**Keywords:** eHealth, medical illustrations, pain perception, mHealth, pain measurement, disease progression, patient-reported outcome measures, musculoskeletal pain, mobile phone, surveys and questionnaires, pain management

## Abstract

**Background:**

Digital pain mapping allows for remote and ecological momentary assessment in patients over multiple time points spanning days to months. Frequent ecological assessments may reveal tendencies and fluctuations more clearly and provide insights into the trajectory of a patient’s pain.

**Objective:**

The primary aim of this study is to remotely map and track the intensity and distribution of pain and discomfort (eg, burning, aching, and tingling) in patients with nonmalignant spinal referred pain over 12 weeks using a web-based app for digital pain mapping. The secondary aim is to explore the barriers of use by determining the differences in clinical and user characteristics between patients with good (regular users) and poor (nonregular users) reporting compliance.

**Methods:**

Patients (N=91; n=53 women) with spinal referred pain were recruited using web-based and traditional in-house strategies. Patients were asked to submit weekly digital pain reports for 12 weeks. Each pain report consisted of digital pain drawings on a pseudo–three-dimensional body chart and pain intensity ratings. The pain drawings captured the distribution of pain and discomfort (pain quality descriptors) expressed as the total extent and location. Differences in weekly pain reports were explored using the total extent (pixels), current and usual pain intensity ratings, frequency of quality descriptor selection, and Jaccard similarity index. Validated e-questionnaires were completed at baseline to determine the patients’ characteristics (adapted Danish National Spine Register), disability (Oswestry Disability Index and Neck Disability Index), and pain catastrophizing (Pain Catastrophizing Scale) profiles. Barriers of use were assessed at 6 weeks using a health care–related usability and acceptance e-questionnaire and a self-developed technology-specific e-questionnaire to assess the accessibility and ease of access of the pain mapping app. Associations between total extent, pain intensity, disability, and catastrophizing were explored to further understand pain. Differences between regular and nonregular users were assessed to understand the pain mapping app reporting compliance.

**Results:**

Fluctuations were identified in pain reports for total extent and pain intensity ratings (*P*<.001). However, quality descriptor selection (*P*=.99) and pain drawing (*P*=.49), compared using the Jaccard index, were similar over time. Interestingly, current pain intensity was greater than usual pain intensity (*P*<.001), suggesting that the timing of pain reporting coincided with a more intense pain experience than usual. Usability and acceptance were similar between regular and nonregular users. Regular users were younger (*P*<.001) and reported a larger total extent of pain than nonregular users (*P*<.001).

**Conclusions:**

This is the first study to examine digital reports of pain intensity and distribution in patients with nonmalignant spinal referred pain remotely for a sustained period and barriers of use and compliance using a digital pain mapping app. Differences in age, pain distribution, and current pain intensity may influence reporting behavior and compliance.

## Introduction

### Background

The term *spinal pain* refers to pain in the cervical, thoracic, and low back areas of the spine [1,2]. Spinal pain can remain localized to the spine and refer to other areas. Cervical and thoracic spinal pain may refer to one or both the upper limbs [2], whereas low back spinal pain may refer to one or both of the lower limbs [2]. Chronic spinal pain is a common reason for clinical consultation [3,4] with an increasing prevalence [5,6] and associated high levels of disability [7].

Pain is a subjective sensory and emotional experience [8], and patients’ self-reported measures of pain, such as pain intensity and quality descriptors, are common during pain assessment [9]. In addition, self-reports of pain distribution can improve the understanding of pain mechanisms [10-12]. Therefore, more efficient patient-clinician communication is paramount to optimize pain management. Traditionally, numerical rating scales capture pain intensity, whereas pain drawings capture pain distribution that is expressed by area (extent) and location [13].

Among the self-reported methodologies, ecological (from the patients’ environment) momentary (in real time) assessment (EMA) methods can help to repeatedly collect information about the patient’s pain condition [14,15]. In the clinical pain field, EMA methods can be used to minimize pain recall bias and assess pain within the patients’ real-world context [14,16] to provide detailed spatiotemporal information and support pain management [12,17-21]. For example, repeated pain intensity reports of patients with musculoskeletal spinal pain may present a stable or fluctuating temporal pattern [22-27]. Similarly, spatiotemporal patterns of pain may present a stable (localized or widespread) or a variable course [28,29]. However, there is little knowledge about the relationship between the course of pain intensity and extent.

Digital biomarkers are defined as objective and quantifiable physiological and behavioral data acquired and measured using digital devices, such as smartphones, tablets, or computers. Thus, digital pain mapping can acquire time-stamped digital pain biomarkers (pain intensity, distribution, and quality). When acquired over multiple time points, a more detailed overview of a patient’s pain experience can be achieved, thereby improving the clinician’s understanding of the patient’s pain [12].

The feasibility of health apps is mostly dependent on its usability and reporting compliance [30,31]. Usability is a broad term that defines the appropriateness of a technology to fit its purpose [32]. A good compliance rate ensures that the requested information is collected [33-38]. However, little is known about the influence of user characteristics and symptom severity on compliance.

### Objectives

This feasibility study aims to map and track pain intensity (current and usual), distribution (extent and location), and quality of pain and discomfort in patients with nonmalignant spinal referred pain for a duration of 12 weeks using a digital pain mapping app. The secondary aim is to determine the barriers to use and individual and clinical factors influencing a patient’s pain reporting compliance using the digital mapping app.

The study aims to address 3 main hypotheses: (1) fluctuations in pain intensity, distribution, and quality over 12 weeks would be identified; (2) pain extent would be associated with current pain intensity and levels of disability; and (3) better reporting compliance would be more likely in patients with more severe pain symptoms.

## Methods

### Overview

This web-based prospective observational cohort study recruited people with nonmalignant spinal referred pain (somatic and radicular) and asked them to submit a weekly pain report for 12 weeks using a web-based pain mapping app. At baseline, all participants completed standardized e-questionnaires about demographics, primary pain site (cervical and thoracic pain or low back pain), disability, and pain catastrophizing. Six weeks into the study, patients were invited to participate in a battery of health care–related usability and acceptance (HUX) e-questionnaire and a self-developed technology-specific e-questionnaire to gain insight into the appropriateness and ease of use of the pain mapping app.

### Participants and Recruitment

The inclusion criteria were kept broad to obtain a general impression of the weekly differences in pain intensity, extent, and quality in patients with nonmalignant spinal referred pain. Men and women (aged 18-85 years) living in Denmark and able to communicate in Danish or English were recruited. Pregnant or breastfeeding women and people with drug abuse and addiction problems were not included. People with a cognitive deficiency, those who lack necessary computer skills (self-assessment or inability to create a password), and/or those who do not have regular internet access were excluded from the study.

In total, 2 recruitment strategies were designed. The traditional strategy consisted of recruiting patients referred to the Neurosurgical Department at Aalborg University Hospital (Denmark). The web-based recruitment strategy consisted of posting a 1-min video on social media platforms (Facebook, LinkedIn, and Twitter) with a call-to-action requesting those interested in taking part in the study to contact the researchers directly via email. Screening was carried out via email correspondence. Additional screening for all patients was carried out by phone interviews, where information about medical history, diagnoses, and current treatment was collected. The patients’ pain management was not monitored or affected during the study.

Patients received detailed information about the study via email, including technical information about registration to the pain mapping app and how to complete the digital pain reports. Subsequently, the researcher (MG) emailed the link to the demographic questionnaire related to their primary pain site (cervical and thoracic or low back). The links to the remaining e-questionnaires (disability and pain catastrophizing) were emailed individually on completion of the previous questionnaire. Each questionnaire took 2 to 3 min to complete.

Furthermore, 6 weeks after the first pain report was submitted, patients completed HUX e-questionnaires, such as the System Usability Scale (SUS) [32] and the modified Technology Acceptance Model (mTAM) questionnaire [39] and a self-developed technology-specific questionnaire.

### Baseline Patient Profile

Baseline e-questionnaires collected information about the patients’ general demographics, walking distance ability, health and social information, disability, and pain beliefs. A maximum of 3 reminders were emailed to the patients, each 1 week apart. If a questionnaire was not completed following the 3 reminders, the patient received a final direct email reminder. Questionnaires were delivered using the SurveyXact software (Ramboll). Permission was obtained to use and adapt all the paper-format questionnaires to the electronic versions used in this study.

The general information questionnaire, adapted from the Danish National Spine Register’s (DaneSpine) basic information questionnaire for degenerative spinal disorders, was used to collect information regarding demographics, walking distance ability, health, and social status. Disability was measured by the Oswestry Disability Index (ODI) in patients whose primary pain site was low back and by the Neck Disability Index (NDI) in patients whose primary pain site was the cervical or thoracic spine. The ODI and the NDI have been used since 1990 and are recommended tools for evaluating self-rated disability in spinal disorders [40,41]. The Pain Catastrophizing Scale (PCS) was used to evaluate pain-related catastrophizing thoughts. Catastrophizing is a negative anticipatory response associated with higher pain intensity [42,43].

### Quantification of Pain Intensity, Extent, and Distribution Consistency

Navigate Pain (Aglance Solutions) allows for the EMA of the temporal development of pain intensity and extent, thereby facilitating an objective and easy visualization of pain changes over time [44,45]. The web-based digital pain mapping app allows users to complete individual pain reports. The pain mapping app had a zoom feature and the option of moving the body chart on the screen to facilitate the visualization and capturing of pain. In each pain report, users indicated their pain area and location on a pseudo–three-dimensional body chart avatar in different views (anterior, posterior, lateral right, and lateral left) and provided a usual and a current pain intensity rating. Usual pain intensity was defined as the pain felt most of the time, whereas current pain intensity was defined as the pain experienced at the time of reporting.

Patients received an email with a link to create a password for accessing the pain mapping app free of charge. Patients used a computer mouse or a touch screen device (ie, smartphone or tablet) to report the distribution of pain and discomfort on a male or female body chart. Patients selected among 11 color-coded pain and discomfort quality descriptors: tingling, throbbing, stabbing, dull aching, numbness, itchy, electric, cold, burning, other, and the general descriptor *pain*. The number of pixels was extracted from the drawn areas in the body charts, including the different views, to quantify the total pain and discomfort extent (total extent). Only one pain report was used to determine the extent and distribution of pain and discomfort each week. If patients submitted more than one weekly pain report, the first pain report following the weekly reminder or the report closest to a 7-day interval was selected. Weekly pain intensity ratings were calculated using all reports for each week.

Patients rated their overall usual and current pain intensity using 2 electronic Color Analogue Scales (eCASs). The eCAS is a colored line (green, yellow, and red) accompanied by the words *no pain*, *moderate pain*, and *severe pain* [46,47].

The consistency of pain distribution may assist in a more objective decision-making process. To assess the consistency of pain and discomfort distribution, 1 pain drawing in the posterior view for each of the 12 weeks was extracted from the weekly pain reports. The similarity of degree of distribution among the weekly pain drawings was calculated and expressed using the Jaccard similarity coefficient, also known as the Jaccard index [48]. The Jaccard indices were calculated between consecutive pain drawings (week to week) during the 12-week period. A higher Jaccard index represents a greater pixel overlap between drawings and is a proxy measure to determine the degree of changes in the distribution (ie, combination of the location and area) of pain and discomfort between weekly pain reports [11,12,48]. Owing to the inconsistent use of the anterior and lateral body chart views, only the posterior view was used to calculate the Jaccard index. The posterior view was used in all the weekly pain reports and was found to be the most relevant view to capture changes in spinally referred pain.

### Assessment of Pain and Discomfort Quality Descriptors

Pain and discomfort quality descriptors are clinically useful during the differential diagnosis process, especially for neuropathic pain [49-52]. The frequency of pain and discomfort quality descriptor selection was calculated by normalizing the number of times a quality descriptor was selected. Therefore, a selected descriptor was only accounted for once weekly for each patient.

### Health Care–Related Usability, Acceptance, and Technology-Specific Questionnaires

To explore the barriers of use, patients who had completed at least one pain report at 6 weeks were deemed as users and invited to complete a battery of electronic questionnaires, including HUX questionnaires and a technology-specific questionnaire. The HUX questionnaires consisted of SUS and mTAM questionnaires to assess the usability and acceptance of the pain mapping app among patients.

The SUS is a simple 10-statement questionnaire evaluating the user experience before any discussion with the researcher. There are alternating positive and negative statements. The version used here replaced the term *the product* to the name of the digital body mapping software *Navigate Pain*. An SUS score over 68 is considered above average [32]. The Technology Acceptance Model (TAM) [39] was modified (mTAM) with the term *digital body chart* to describe the technology. The mTAM consisted of a total of 9 statements—4 of these rated perceived usefulness and 5 rated perceived ease of use. Both questionnaires use a 5-point Likert scale, ranging from 1 for *strongly disagree* to 5 for *strongly agree*.

A Navigate Pain Specific (NPS) questionnaire was developed to examine user behavior, particularly accessibility and ease of access. Patients were asked to answer 5 questions. Of these questions, 2 questions assessed point of access to the mapping app (computer or laptop, tablet, or smartphone) and the access pathway to the pain mapping app (reminder email or direct URL link). One question used a 5-point Likert scale, ranging from 1 for *very helpful* to 5 for *not at all helpful*, to explore the helpfulness of the weekly pain report reminder. The last 2 questions also used a 5-point Likert scale, ranging from 1 for *very easy* to 5 for *very difficult* to assess the registration process and the general patients’ perspective of ease of use.

### Differences Between Regular and Nonregular Users

Patient characteristics were assessed in relation to pain reporting compliance. Regular users completed weekly pain reports, whereas nonregular users did not fulfill this criterion. In the NPS questionnaire, nonregular users received an additional question asking for reasons for the lack of regular pain reporting, such as “I didn’t have time,” “I forgot to do it,” “I am in too much pain to do it,” “I don’t have pain,” “I’m not interested in using Navigate Pain any more,” and “Other.”

### Statistical Analysis

Descriptive statistics were used to describe demographics and baseline characteristics and quality descriptor selection. Data distribution was assessed with histograms and quantile-quantile plots for pain intensity ratings, total extent (pixels), and pain distribution (Jaccard index). For nonparametric data, Friedman tests were used to assess the pain intensity ratings (usual and current) and the total extent between weeks. A Wilcoxon signed-rank test was used to compare the differences between current and usual pain intensity ratings. The consistency of pain distribution was determined by comparing the Jaccard indices each week using a Friedman test. A logistic multiple regression analysis was carried out to explore the influence of reporting compliance on pain distribution consistency. A chi-square test was used to assess differences in weekly pain and discomfort quality descriptor selection during the 12 weeks.

Spearman correlation was used to determine the associations between the pain intensity ratings and the total extent for each patient and the baseline disability and PCS scores.

Mann-Whitney *U* tests were used to examine the differences in baseline characteristics between regular users and nonregular users, such as age, gender, primary pain site, usual and current pain intensity, total extent, disability, PCS scores, and HUX and NPS questionnaire results. A chi-square test was used to explore recruitment differences between regular and nonregular users. A logistic multiple regression analysis was carried out to explore the probability of better reporting compliance in relation to the severity of the pain symptoms. The assessment of pain severity included current pain intensity ratings, extent of pain quality (posterior view) from the first submitted pain report, and baseline disability (ODI or NDI) scores.

Statistical analyses were performed using SPSS 25 (SPSS Statistics, 2018). The Jaccard indices were calculated using MATLAB R2017b (The MathWorks, Inc). Correlation coefficients, means, SD values, median, and IQR were reported where relevant. *P* values of less than .05 were considered statistically significant, and Bonferroni adjustment was used in multiple analyses.

In Denmark, observational studies using surveys and questionnaires are not required to obtain ethical approval but are required to be registered with the Danish Data Protection Agency (journal numbers 2017-899/10-0159 and 2017-509-00011). This study adhered to ethical data privacy and storage General Data Protection Regulation requirements and was conducted according to the Declaration of Helsinki. The study is registered with ClinicalTrials.gov (NCT03926364).

## Results

### Baseline Patient Profile

**Figure 1:**
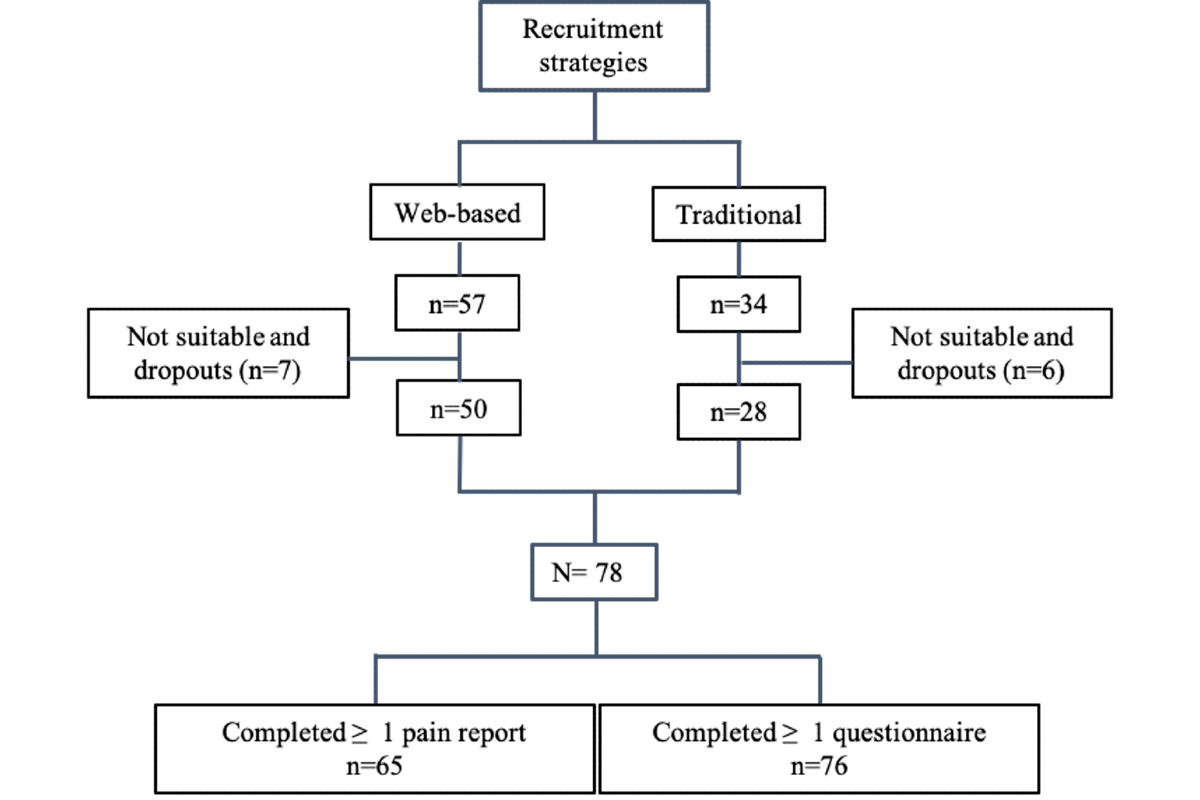
CONSORT (Consolidated Standards of Reporting Trials) flow diagram, showing the complete recruitment process.

A total of 91 patients (mean age 51.8, SD 13.5 years; 53 women) were willing to participate in the study. Recruitment methods included a traditional in-house strategy and a web-based strategy. Following screening and early dropouts, a total of 78 patients were recruited to participate in the study (Figure 1). Overall, 92% (12/13) of the early dropouts were due to technical difficulties with creating a password that limited access to the pain mapping app.

Overall, 64% (50/78) of the participants were recruited using the web-based strategy. Only an age difference was identified between the patients recruited through the web-based strategy (mean age 48.7, SD 12.1 years) and the traditional in-house strategy (mean age 59.2, SD 13.4 years; *P*<.001).

A total of 3863 pain reports were submitted (mean 59, SD 66, per patient). All the patients recruited for this study had pain for longer than 6 months, with 79% (60/76 completed questionnaires) of patients having pain for over 12 months (Table 1), thus fulfilling the criteria of chronic or persistent pain. Walking ability distance, disability, and catastrophizing scores at baseline are shown in Table 2.

**Table 1:** Self-reported patients’ baseline characteristics.^a^

Demographics		Values			
		n (%)		Mean (SD)	
Age (years)		76 (100)		51.78 (13)	
**Gender**					
	Male		24 (32)		N/A^b^
	Female		52 (68)		N/A
**Primary pain site**					
	Cervical and thoracic		17 (22)		N/A
	Low back		59 (78)		N/A
BMI (kg/m^2^)		76 (100)		27.69 (5.3)	
Regular smokers		10 (12)		N/A	
**Regular alcohol intake**		21 (27)		N/A	
	Units per week		N/A		8.3 (6.3)
**Current social history**					
	Retired due to spinal pain		19 (25)		N/A
	Currently on sick leave		19 (25)		N/A
**Pain duration**					
	Between 3-12 months		16 (21)		N/A
	Between 12-24 months		9 (12)		N/A
	More than 24 months		51 (67)		N/A
**Regular analgesia intake**		43 (75)		N/A	
	Morphine		22 (39)		N/A
**Self-reported diagnoses**					
	Number of patients		61 (80)		N/A
	Discus prolapse or protrusion		28 (63)		N/A
	Degenerative changes		14 (32)		N/A
	Nonspecific		14 (32)		N/A
	Spinal stenosis		13 (30)		N/A
	Modic changes		10 (23)		N/A
	Spondylosis		7 (9)		N/A
	Scoliosis		6 (8)		N/A
	Spondylolisthesis		2 (3)		N/A
	Hypermobility		1 (1)		N/A
	Chronic pelvic pain		1 (1)		N/A
**Past medical history**					
	Cardiac condition		4 (5)		N/A
	Neurological condition		8 (10)		N/A
	Cancer		0 (0)		N/A
	Other (painful condition)		20 (26)		N/A
	Other (affecting mobility)		17 (22)		N/A
**Previous spinal surgery**					
	Once		11 (15)		N/A
	Twice		5 (7)		N/A
	More than twice		4 (5)		N/A

^a^The self-reported diagnoses include all the diagnoses reported by the patients.

^b^N/A: not applicable.

**Table 2:** Baseline walking distance ability, disability, and catastrophizing scores.

Walking distance and disability				Values			
				n (%)		Mean (SD)	
**Walking distance ability**							
	<100 m			9 (12)		N/A^a^	
	100-150 m			12 (16)		N/A	
	0.5-1 km			7(10)		N/A	
	More than 1 km			48 (62)		N/A	
**Oswestry Disability Index**				46 (78)		35.16 (15.9)	
	Minimal disability			11 (24)		N/A	
	Moderate disability			19 (41)		N/A	
	Severe disability			15 (33)		N/A	
	Crippled			1 (2)		N/A	
**Neck Disability Index**				19 (100)		34.7 (19.9)	
	No disability			0 (0)		N/A	
	Mild disability			3 (16)		N/A	
	Moderate disability			4 (21)		N/A	
	Severe disability			3 (16)		N/A	
	Complete disability			9 (47)		N/A	
**Catastrophizing beliefs**							
	**Pain Catastrophizing Scale**			51 (67)		21.92 (12.3)	
		Rumination		N/A		7.4 (4.7)	
		Magnification		N/A		4.11 (3.06)	
		Helplessness		N/A		10.41 (12.3)	
		Total score >30		15 (20)		N/A	

^a^N/A: not applicable.

### Quantification of Pain Intensity, Total Extent, and Distribution Consistency

**Figure 2:**
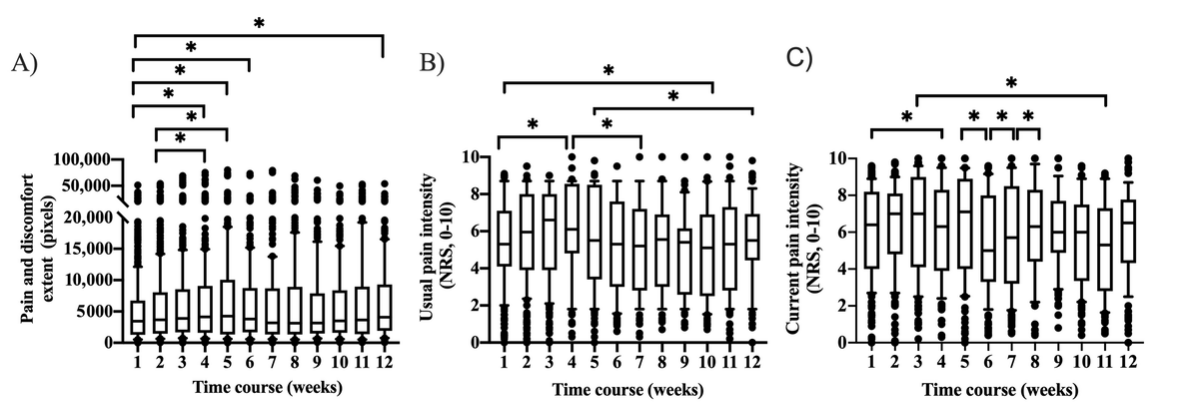
Median (IQR) pain and discomfort extent (A) and usual (B) and current (C) pain intensity ratings, increased and decreased as a group (n=65), in comparison with baseline and throughout the 12 weeks. The lower and upper quartiles, representing observations between the 25th and 75th percentile range, show the median for a month. The whiskers are drawn down to the 10th percentile and up to the 90th. Points below and above the whiskers are drawn as individual dots. *P<.001 adjusted for multiple comparisons. NRS: numerical rating scale.

Current pain intensity ratings (median 6.3, IQR 4.5) were greater than usual pain intensity ratings (median 5.4, IQR 4.0; Z=−18.0; *P*<.001) when compared over the 12 weeks. Furthermore, fluctuations were identified in terms of usual pain intensity ratings (*χ^2^*_11_=145.3; *P*<.001), current pain intensity ratings (*χ^2^*_11_=105.7; same *P*<.001), and total extent (*χ^2^*_11_=48.7; *P*<.001) over the 12 weeks (Figure 2). Comparisons of subsequent pain drawings showed similar Jaccard indices (*P*=.52) for the study cohort. The logistic regression model was not statistically significant (*χ^2^*_11_=8.1; *P*=.70).

### Frequency of Pain and Discomfort Descriptors

**Figure 3:**
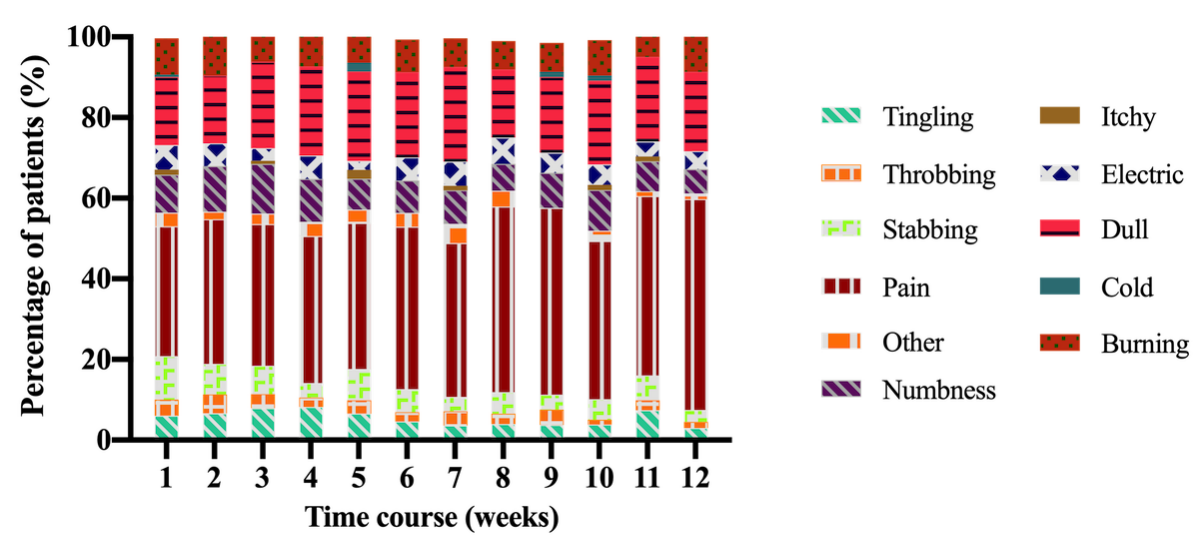
The most frequently selected pain and discomfort quality descriptors over 12 weeks were pain (39%) and dull (20%). The qualities numbness (9%), burning (8%), and stabbing (6%) were also chosen frequently. The least frequently selected quality descriptors were throbbing (3.2%), other (2.8%), itchy (0.8%), and cold (0.4%).

**Figure 4:**
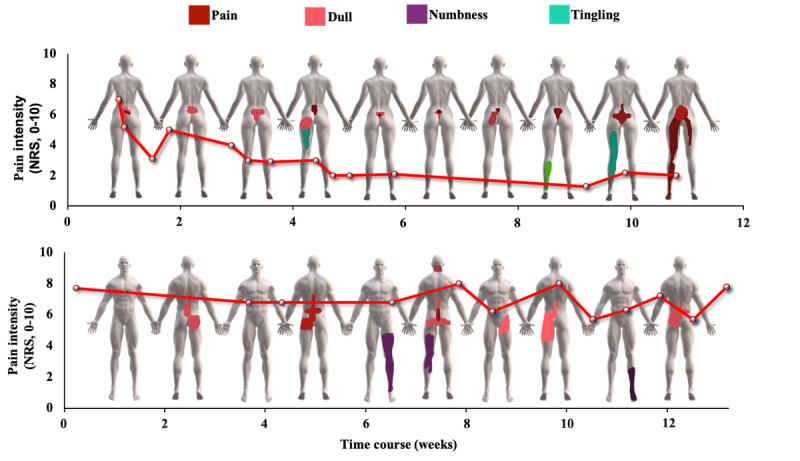
Examples of individual pain reports illustrating fluctuations in pain and discomfort intensity, total extent, and quality, spanning 12 weeks. The red line represents the weekly average current pain intensity rating. The pain drawings were selected every second week to capture the general overview of the changes in pain and discomfort quality descriptors selection and the fluctuations in pain and discomfort extent over the 12 weeks. NRS: numerical rating scale.

A chi-square test revealed that the selection of quality descriptors remained stable over the 12 weeks (*P*=.99; Figure 3). However, individual variations in pain and discomfort quality descriptor selection were observed (Figure 4).

### Relationship Among Pain Intensity, Total Extent, Disability, and Pain Catastrophizing

Usual and current pain intensities were associated with the total extent (*r*=0.23 and *r*=0.25, respectively; *R*^2^=3%; *P*<.001). Disability, as rated by the ODI and NDI, was not associated with the current and usual pain intensities (*P*=.29 and *P*=.09, respectively), total extent (*P*=.31), and pain catastrophizing (*P*=.83). Similarly, pain catastrophizing was not associated with usual and current pain intensities (*P*=.89 and *P*=.71, respectively) and the total extent (*P*=.29).

### Differences Between Regular and Nonregular Users

Regular users (n=35; mean age 48.7, SD 11.2 years) and nonregular users (n=30; mean age 55.8, SD 15.3 years) differed in age (*P*<.001). Interestingly, nonregular users had more intense current pain (median 6.4, IQR 3.2) than the regular users (median 6.1, IQR 5.1; *U*=3306.7; *P*<.001). However, regular users had a greater total extent (regular users 4063, IQR 8075.5; nonregular users 3221, IQR 4925.0; *U*=2,775,320.5; *P*<.001). In addition, 80% (28/35) of the regular users were recruited through the web, as opposed to 56% (17/30) of the nonregular (*χ^2^*_2_=28.9; *P*<.001). Current pain intensity, pain extent, and disability did not influence reporting compliance between regular and nonregular users (*P*=.96).

### HUX Analyses

HUX questionnaires were completed by 94% (33/35) of the regular and 73% (22/30) of the nonregular users. The total mean SUS score was 70, giving it a marginal usability score [53] with no difference between regular and nonregular users (*P*=.45). Regular users rated acceptable usability [54] with a mean SUS score of 71.5 points, whereas nonregular users rated marginal usability [53] with a mean SUS score of 68 points. There were no differences in mTAM scoring between the regular and nonregular users, with both groups having similar acceptance scores in each of the questionnaire statements (*P* value range for each statement=.12-.97).

Of the regular users, 49% (17/35) accessed the pain mapping app from a computer or laptop and did not need the weekly reminder to submit the pain reports; nonregular users accessed the pain mapping app using a variety of devices, and 82% (20/30) depended on the weekly reminder to submit the pain reports. Furthermore, 45% (10/30) and 41% (9/30) of the nonregular users reported forgetfulness and *other* as the reasons for poor reporting compliance. The remaining 13% of nonregular users reported too much (2/30) or no pain (1/30). None of the patients selected *I am not interested anymore* or *I did not have time*.

Regular users rated the general use of the pain mapping app as easy or very easy (15/35, 45% and 8/30, 27%, respectively). Similarly, nonregular users rated the general use of the pain mapping app as *easy* and *very easy* (11/30, 50% and 4/30, 18%), respectively. Only 6% (6/35) of the regular and 9% (2/30) of the nonregular users rated the general use of the pain mapping app as *difficult.*

## Discussion

### Principal Findings

This is the first pragmatic observational study using a web-based app to map, track, and quantify pain and discomfort remotely in patients with nonmalignant spinal referred pain over a sustained period. The results show fluctuations in current and usual pain intensities over 12 weeks for the group of patients recruited for this study. In addition, the results show fluctuations in pain and discomfort extent (total extent) on a group level. However, the pain drawings and the quality descriptor selection remained consistent on a group level. HUX scores were similar between regular and nonregular users. The regular users were generally younger, had a greater total extent, and accessed the app differently than the nonregular users.

Fluctuations in current and usual pain intensities and in total extent occurred over the 12-week observation period, suggesting that spatiotemporal patterns of chronic spinal referred pain may increase and decrease over time. These fluctuations may be related to the heterogeneity of the study cohort in which participants had different primary pain sites, differences in pain management, and differences in reporting compliance. Thus, there is no rationale for why the group pain intensity and total extent varied on any given week over the 12-week observation period. An interesting observation in this study was that current pain intensity ratings were greater than usual pain intensity ratings, suggesting that patients completed pain reports when their pain was more intense than usual. The reasons for this reporting behavior are unclear and should be further explored. However, the difference between current and usual pain intensity ratings was small (<1 of 10) and may not be clinically meaningful [54].

The selection of pain and discomfort quality descriptors remained consistent over time, which can be expected for a group with chronic pain. Interestingly, pain distribution also remained consistent. In this study, pain distribution consistency was calculated by comparing the similarity of consecutive weekly pain drawings. Therefore, the study’s results do not imply that pain distribution was consistent throughout the 12-week study period. It is possible that fluctuations in pain distribution may occur over longer periods (Multimedia Appendix 1). The Jaccard index has primarily been used to assess the ability of patients to reliably draw and redraw pain areas [48,55]. Therefore, the Jaccard index may only be appropriate to assess distribution consistency over shorter periods.

The study’s findings align with previous studies [56-59] showing a weak and positive association between the pain intensities (usual and current) and the total extent. These results suggest that pain extent is related to pain intensity but do not explain the intensity variance. Therefore, capturing changes in clinical pain extent and distribution may add additional value to clinical interpretation.

The relationships of pain intensity and extent with disability and catastrophizing scores are contradictory. Similar to our study, studies on chronic low back pain [60,61] and knee osteoarthritis [59] have shown that pain intensity and total extent were not associated with disability and pain catastrophizing. However, other studies have shown a positive relationship of the total extent with disability scores [11,62-66] and pain catastrophizing [43,62,67-69]. The relationship between catastrophizing and disability scores has been widely described in musculoskeletal shoulder and low back pain [70-72]. However, patients in our study reported high levels of disability but not high levels of catastrophizing, showing no association between disability and catastrophizing at baseline.

The HUX results demonstrated that the pain mapping app was considered a good pain communication tool by patients and therefore may be relevant for clinical practice. However, the HUX assessment failed to identify compliance differences, as represented by the similar results between regular and nonregular users.

Most of the regular users were recruited using the web-based strategy. Patients recruited through the web were younger (approximately by 3 years) and may already be more technically competent [73] than those recruited using the traditional strategy. A limited number of studies have assessed users’ characteristics influencing the use and acceptance of pain technology [74]. Overall, regular users were younger (approximately by 7 years) and reported a larger pain extent than nonregular users, suggesting that reporting compliance of the pain mapping app may be based on an inherent need to communicate the pain extent [75]. Interestingly, the total extent differences between regular and nonregular users (approximately 850 pixels) represents a very small area on a pain drawing and may be insufficient to be clinically relevant. In a short period, the 7-year age gap between regular and nonregular users will become irrelevant as technical savviness increases among older adults [76]. Therefore, it is possible that in the future, only relevant baseline differences influencing compliance may be based on the recruitment method.

### Limitations

EMA can induce bias because of the lack of standardization of pain reporting in terms of context (location and environment), timing, and frequency [15,77]. This study lacks contextual information about, for example, type or change in pain management and activity levels. Therefore, it is not known whether the fluctuations in total extent reporting may be due to a change in experienced pain or a change in the number of pain report submissions over time. Furthermore, fluctuations could also be due to a change in the drawing skills, as the patients’ drawing confidence may have increased with repetition or even whether patients used different accessing devices affecting their technical skills.

Technical limitations influenced the total extent results and the distribution consistency index. First, the total extent may be overestimated, as it is the sum of all the quality descriptors used in a pain report. Second, only the Jaccard index was used as a measure of distribution consistency. Using one single index, the Jaccard index, to measure pain distribution consistency, carries potential risks for misinterpretation, as it will miss similarities between 2 pain drawings with equal pain areas but different locations, representing a small analytical variation, but with clinical implications.

Technical and interpretation barriers of use were identified. Technical barriers caused most of the early dropouts, likely because of using an old version of a browser, and may have biased the usability ratings. Interpretation barriers led to different drawing behaviors despite receiving the same instructions, highlighting different ways of understanding the provided pain reporting instructions or perceiving the individual pain experience (Multimedia Appendix 2).

### Future Perspectives

The digital pain biomarkers acquired from the app can assist in a more objective diagnostic process and monitor the outcomes following treatment. An example of outcome monitoring could be useful following spinal surgery or a conservative approach, such as exercise therapy, where the pain mapping app can be used to monitor changes in pain intensity and referred arm and leg pain distribution [78]. New metrics for assessing consistency over a longer period may prove clinically valuable as fluctuating and stable pain reports may require different pain management approaches.

The usefulness and advantages of digital pain mapping to track digital pain biomarkers, combined with machine learning, have already revealed spatiotemporal patterns of pain and discomfort [10]. These patterns have the potential to become a game changer and may be able to predict those patients more likely to respond to treatments or assist in the prognosis of pain conditions [79].

### Conclusions

This is the first study to remotely track pain intensity and distribution and examine barriers of use and compliance using a digital mapping pain app for a sustained period. Differences in age and pain distribution may influence reporting behavior and compliance and recruitment strategies that may play a role in the success of future web-based studies.

## Appendix

Multimedia Appendix 1 Jaccard indices for each individual patient (n=59) with nonmalignant chronic spinal referred pain in cervical or thoracic and low back (gray) and the group average (black) showing the degree of similarity between 2 consecutive weekly pain and discomfort drawings over the 12 weeks.

Multimedia Appendix 2 Example of individual pain drawings showing different drawing behaviors following the delivery of the same instructions.

## Abbreviations

eCAS: electronic Color Analogue Scale
EMA: ecological momentary assessment
HUX: health care–related usability and acceptance
mTAM: modified Technology Acceptance Model
NDI: Neck Disability Index
NPS: Navigate Pain Specific
ODI: Oswestry Disability Index
PCS: Pain Catastrophizing Scale
SUS: System Usability Scale
TAM: Technology Acceptance Model
