# Supplementary figures and images for: Digital Pain Mapping and Tracking in Patients With Chronic Pain: Longitudinal Study
Source: J Med Internet Res. 2020 Oct 26;22(10):e21475. doi: 10.2196/21475 (PMC7652695; doi:10.2196/21475)

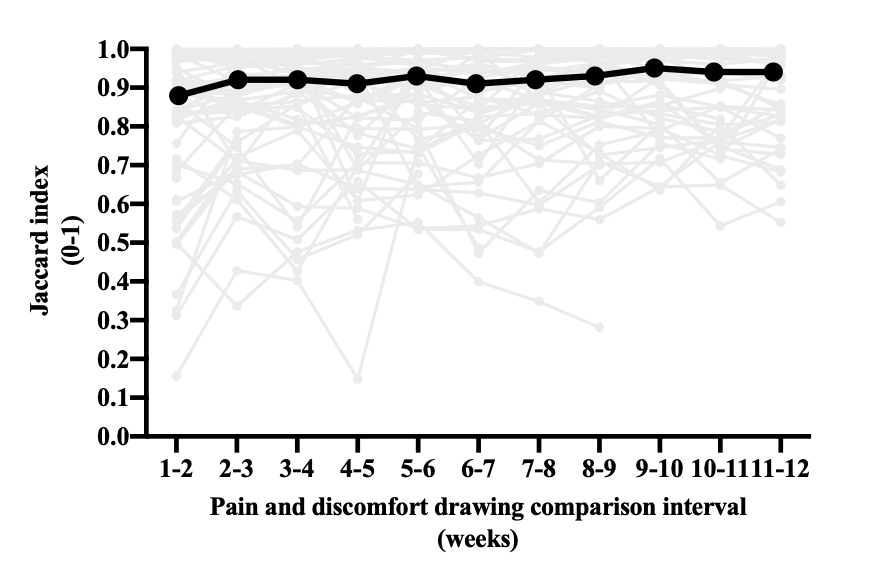

Supplement: Multimedia Appendix 1 [file jmir_v22i10e21475_app1.png]

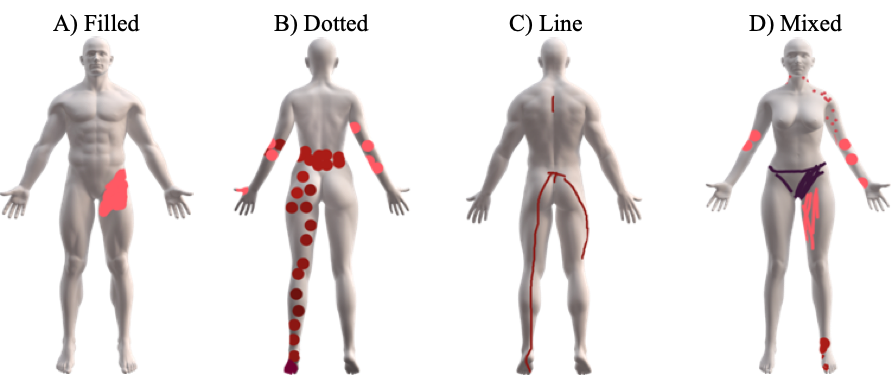

Supplement: Multimedia Appendix 2 [file jmir_v22i10e21475_app2.png]
